# Behavioral and Neural Variability of Naturalistic Arm Movements

**DOI:** 10.1523/ENEURO.0007-21.2021

**Published:** 2021-06-19

**Authors:** Steven M. Peterson, Satpreet H. Singh, Nancy X. R. Wang, Rajesh P. N. Rao, Bingni W. Brunton

**Affiliations:** 1 Department of Biology, University of Washington, Seattle, Washington 98195; 2 eScience Institute, University of Washington, Seattle, Washington 98195; 3 Department of Electrical & Computer Engineering, University of Washington, Seattle, Washington 98195; 4 IBM Research, San Jose, California 95120; 5 Paul G. Allen School of Computer Science and Engineering, University of Washington, Seattle, Washington 98195; 6 Center for Neurotechnology, University of Washington, Seattle, Washington 98195

**Keywords:** electrocorticography, naturalistic neuroscience, spectral power

## Abstract

Motor behaviors are central to many functions and dysfunctions of the brain, and understanding their neural basis has consequently been a major focus in neuroscience. However, most studies of motor behaviors have been restricted to artificial, repetitive paradigms, far removed from natural movements performed “in the wild.” Here, we leveraged recent advances in machine learning and computer vision to analyze intracranial recordings from 12 human subjects during thousands of spontaneous, unstructured arm reach movements, observed over several days for each subject. These naturalistic movements elicited cortical spectral power patterns consistent with findings from controlled paradigms, but with considerable neural variability across subjects and events. We modeled interevent variability using 10 behavioral and environmental features; the most important features explaining this variability were reach angle and day of recording. Our work is among the first studies connecting behavioral and neural variability across cortex in humans during unstructured movements and contributes to our understanding of long-term naturalistic behavior.

## Significance Statement

Understanding the neural basis of human movement has long been a key focus in neuroscience. However, researchers often study constrained, monotonous tasks that differ greatly from the rich and diverse natural movements we actually make. Here, we use data-intensive computational approaches to reveal patterns in neural activity underlying naturalistic human arm movements. While such movements match previous experimental findings on average, there is substantial neural variability from one movement to the next. We partially explain this variability by aspects of the movement observed, though much variability remains unaccounted for. Our study sheds light on how the brain generates natural arm movements and emphasizes the critical need to study brain activity in more unstructured settings that mimic daily life.

## Introduction

Natural human movements are remarkable in their complexity and adaptability, relying on precisely coordinated sensorimotor processing in several cortical regions (Miller et al., 2007; Truccolo et al., 2008; Kalaska, 2009; Sober et al., 2018). Much of our understanding on the neural basis of human upper-limb movements has been gained by studying constrained, repetitive movements in the laboratory, using paradigms such as the center-out reaching task (Leuthardt et al., 2004; Georgopoulos et al., 2007; Schalk et al., 2008; Wang et al., 2012; Nakanishi et al., 2013). Center-out reaching is an elegant method for investigating the neural basis of movement, but it remains unclear how well its findings generalize to the spontaneous, unstructured actions observed in the real world (Fried et al., 2017; Umeda et al., 2019). Studies have enhanced the realism of experimental reaching paradigms by incorporating self-cued and less restrictive movements (Romo and Schultz, 1987; Lee and Assad, 2003; Jackson et al., 2007; Kornhuber and Deecke, 2016), but few studies have focused on completely unstructured, naturalistic human movements recorded outside of defined laboratory paradigms. Focusing on such naturalistic behavior enriches our understanding of the relationship between motor behavior and cortical activation (Dastjerdi et al., 2013) and motivates development of robust brain–computer interfaces to restore impaired movement and sensation across diverse contexts (Taylor et al., 2002; Schalk et al., 2008; Gilja et al., 2011; Omedes et al., 2018; Wilson et al., 2019).

Intracranial electrophysiological recordings offer a unique view into the neural correlates of human behavior. These recordings, obtained using electrocorticography (ECoG), contain physiologically relevant spectral power patterns corresponding to a variety of behaviors (Pistohl et al., 2008; Gunduz et al., 2011; Takaura et al., 2016; Anumanchipalli et al., 2019; Miller, 2019). ECoG recording electrodes are implanted on the cortical surface, beneath the skull and dura; these signals are thus cleaner and less susceptible to artifact contamination than signals from electroencephalography (EEG; Ball et al., 2009). Although implanting ECoG electrodes is an invasive neurosurgical procedure, the recordings are highly informative and have a combination of high spatial and temporal resolution not found in other human neuroimaging or neural recording modalities (Jacobs and Kahana, 2010; Schalk and Leuthardt, 2011; Kanth and Ray, 2020).

During instructed upper limb movements, ECoG spectral power in frontoparietal cortical areas, particularly over sensorimotor cortex, has been shown to transiently increase at high frequencies and decrease at low frequencies (Miller et al., 2007; Pistohl et al., 2012; Talakoub et al., 2015, 2017). Similar spectral power changes have also been observed in EEG and local field potential recordings across a wide variety of movement behaviors (Milekovic et al., 2015; Ofori et al., 2015; Tan et al., 2016; Chung et al., 2018; Peterson and Ferris, 2018). An important attribute of ECoG recordings is that the patients are being continuously monitored over long periods of time, often approximately a week, providing unique opportunities to collect long-term datasets during unconstrained, uninstructed movements (Chao et al., 2010; Vansteensel et al., 2013; Wang et al., 2016, 2018; Alasfour et al., 2019; Gabriel et al., 2019). However, the behavioral and neural variability of such spontaneous, naturalistic movements remains unexplored.

Analyzing naturalistic data presents formidable challenges, but recent innovations in data science make it possible to extract meaningful findings from increasingly complex, including naturalistic and opportunistic, datasets (Brunton and Beyeler, 2019). Without prior experimental design or direct behavioral measurements, a critical first step in analyzing naturalistic data had previously been laborious manual annotation of behavior. Such tedious labeling severely limits the amount of usable data and is prone to subjective error. Fortunately, recent advances in computer vision and machine learning have enabled substantial automation of the analysis and quantification of naturalistic behaviors (Anderson and Perona, 2014; Berman, 2018; Brown and de Bivort, 2018; Mathis et al., 2018; Datta et al., 2019). Even so, making sense of annotated behavior remains challenging in the absence of a controlled experimental paradigm. There are often many possible ways to characterize behavioral features, making it challenging to select ones that are objective and neurally relevant. Fortunately, previous upper-limb movement studies have identified several neurally relevant behavioral features that can be obtained without subjective, manual identification. Such behavioral features include the angle, duration, magnitude, and velocity of the movement as well as whether or not the movement was bimanual (Donchin et al., 2002; Heldman et al., 2006; Ebner et al., 2009; Mooshagian et al., 2018). Based on previous research (Dumas et al., 2010; Derix et al., 2012), we were also motivated to consider the effects of social interaction on neural activity. Finally, ECoG recordings are nonstationary (Klosterman et al., 2016; Yang et al., 2017), so we considered how movement-related neural activity varied over several hours and across recording days.

In this article, we analyzed opportunistic, clinical intracranial recordings from 12 human subjects across 3–5 d each as we observed their naturalistic spontaneous arm movements. We developed an automated approach to identify and characterize thousands of spontaneous arm movements, enabling scalable analysis of video that was acquired simultaneously with the intracranial recordings. We characterized the variability of both naturalistic upper-limb reaching movements and the corresponding changes in cortical spectral power. Based on findings from controlled experiments, we hypothesized that naturalistic reaches would be associated with transient low-frequency power decreases and high-frequency power increases, localized to frontoparietal sensorimotor cortices (Miller et al., 2007; Talakoub et al., 2017). We also assessed the variability of these event-related spectral power fluctuations and performed regression to partially explain this neural activity using behavioral features.

## Materials and Methods

### Subject information

We analyzed opportunistic clinical recordings from 12 subjects (8 males, 4 females) during their clinical epilepsy monitoring. Subjects were (mean ± SD) 29.4 ± 7.9 years old at the time of recording. Our study was approved by the University of Washington Institutional Review Board for the protection of human subjects. All subjects provided written informed consent.

We selected subjects who had ECoG electrode coverage near primary motor cortex, with either one 8 × 8 or two 4 × 8 electrode grids placed subdurally on the cortical surface. Additional electrodes were implanted on the cortical surface for some subjects, resulting in 87.0 ± 12.9 total surface electrodes per subject (mean ± SD). In addition, five subjects had 23.2 ± 12.1 intracortical depth electrodes (mean ± SD). Electrodes were implanted primarily within one hemisphere for each subject (five right hemisphere, seven left hemisphere). Single-subject electrode placement and recording duration information are given in Extended Data Figure 1-1.

**Figure 1.**
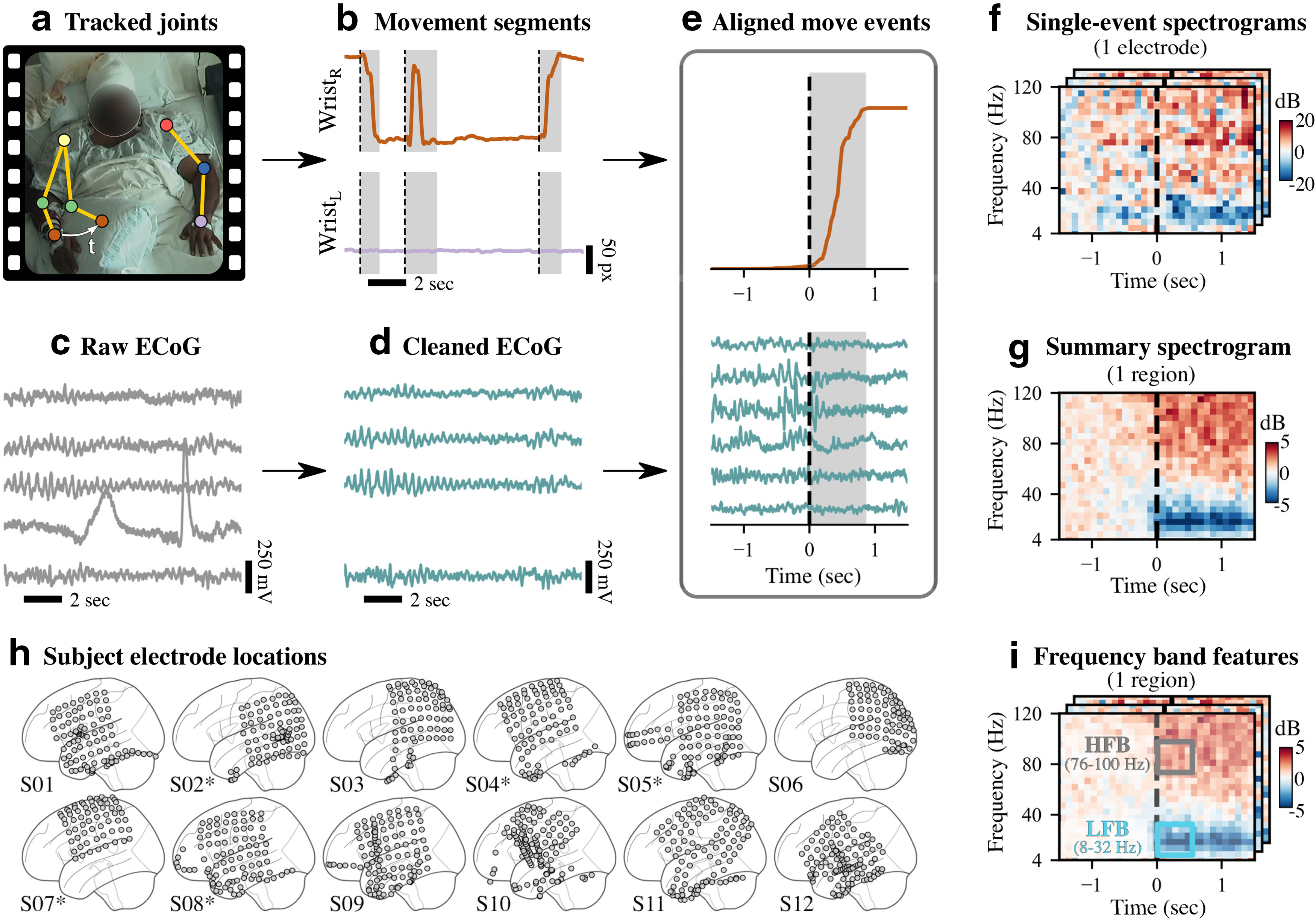
Schematic overview of data-processing, analysis, and modeling framework. ***a***, ***b***, Based on continuous video monitoring of each subject (example video frame shown in ***a***), trajectories of the left and right wrists (Wrist_L_ and Wrist_R_ in ***b***) were estimated using neural networks (Mathis et al., 2018) and automatically segmented into move (gray) and rest (white) states as shown in ***b***. ***c***, ***d***, Raw multielectrode ECoG was filtered and rereferenced; bad electrodes (e.g., ones with artifacts) were removed from further analysis. ***e***, Movement onset events detected from video as shown in ***b*** were aligned with ECoG data using time stamps. ***f***, For each move event at each electrode, spectral power was computed and visualized as a log-scaled spectrogram. ***g***, Summarizing across events and electrodes, we projected the spectral power from electrodes onto eight cortical regions based on anatomic registration and computed the median power across movement events. ***h***, Our data included 12 subjects; their electrode placements are shown in MNI coordinates (see Extended Data Figure 1-1 for subject-specific details). Five of the subjects had electrodes implanted in their right hemispheres (denoted by asterisks). For consistency of later analyses, we mirrored these electrode locations as shown. ***i***, To partially explain the event-by-event neural variability in LFB (8–32 Hz) and HFB (76–100 Hz) spectral power, we fit multiple linear regression models at each electrode using behavioral features extracted from the videos.

10.1523/ENEURO.0007-21.2021.f1-1 Figure 1-1 Single-subject recording days used and electrode information. Surface electrodes refer to grid and strip electrodes placed on the cortical surface, while depth electrodes reach deep cortical and subcortical areas. Download Figure 1-1, EPS file.

### Data collection

Subjects underwent 24 h clinical monitoring, involving semicontinuous ECoG and audio/video recordings over 7.4 ± 2.2 d per subject (mean ± SD). Some breaks occurred throughout monitoring [on average, 8.3 ± 3.2 total breaks per subject, each lasting 1.9 ± 2.4 h (mean ± SD)]. For all subjects, we restricted our analysis to days 3–7 following the electrode implantation surgery, to exclude potentially anomalous neural and behavioral activity immediately following electrode implantation surgery. For several subjects, some days were excluded because of corrupted or missing data files, as noted in Extended Data Fig. 1-1. During clinical monitoring, subjects were observed during a variety of typical everyday activities, such as eating, sleeping, watching television, and socializing while confined to a hospital bed. ECoG and video were initially sampled at 1000 Hz and 30 frames per second, respectively. Figure 1 shows an example of the clinical monitoring setup, along with our data-processing pipeline.

### ECoG data processing

We processed the raw ECoG data using custom MNE-Python scripts (Gramfort et al., 2013). First, we removed DC drift by subtracting the median voltage of each electrode. Widespread, high-amplitude artifacts were then identified by abnormally high electrode-averaged absolute voltage [interquartile range (IQR), >50]. We set these artifacts to 0, along with all data within 2 s of each identified artifact. Removing such high-amplitude discontinuities minimizes subsequent filtering artifacts because of large, abrupt changes in the signal (Gibbs, 1899).

With data discontinuities removed, we bandpass filtered the data (1–200 Hz) and notch filtered to minimize line noise at 60 Hz and its harmonics. The data were then resampled to 500 Hz and rereferenced to the common median for each grid, strip, or depth electrode group. Electrodes with bad data were identified based on abnormal SD (IQR, >5) or kurtosis (IQR, >10) compared with the median value across channels. This process resulted in the removal of 4.9 ± 4.9 surface electrodes per subject and 1.0 ± 1.4 depth electrodes for each of the five subjects with depth electrodes.

Electrode positions were localized using the Fieldtrip toolbox in MATLAB (Oostenveld et al., 2011; Stolk et al., 2018) to enable multisubject analyses. This process involved coregistering preoperative MRI and postoperative computed tomography scans, manually selecting electrodes in 3D space, and warping electrode positions into Montreal Neurological Institute (MNI) space.

### Movement event identification and pruning

We performed markerless pose estimation on the raw video footage separately for each subject to determine wrist positions (Fig. 1*a*). First, for each subject, we manually annotated 1000 random video frames with the 2D positions of the following nine key points: the subject’s nose, ears, wrist, elbows, and shoulders (https://tinyurl.com/human-annotation-tool). Video frames were randomly selected across all days, with preference given to frames during active, daytime periods. Note that these annotated frames comprised 0.01% of all video frames for each subject, demonstrating the infeasibility of annotating all video frames by hand. These manually annotated frames were used to train a separate neural network model for each subject using DeepLabCut (Mathis et al., 2018). Each model was then applied to every video for that subject to generate estimated wrist trajectories.

Movement states were identified by applying a first-order autoregressive hidden semi-Markov model to each wrist trajectory. This state segmentation model classified the wrist trajectory into either a move or rest state. For this study, we focused on movements of the wrist contralateral to the implanted hemisphere. Contralateral wrist states were then discretized, and movement initiation events were identified at state transitions where 0.5 s of rest states are followed by 0.5 s of move states (Singh et al., 2020).

After identifying movement initiation events, we coarsely labeled the video data manually (∼3 min resolution) and excluded arm movements during sleep, unrelated experiments, and private times (as specified in our Institutional Review Board protocol). In addition, we only retained movement events where (1) movement durations were between 0.5–4 s; (2) the confidence scores from DeepLabCut were >0.4, indicating minimal marker occlusion; and (3) wrist movements followed a parabolic trajectory, as determined by a quadratic fit to the wrist’s radial movement ($R^{2}>0.6$). We found that this quadratic fit criteria eliminated many outliers with complex movement trajectories and improved the interpretability of our subsequent analyses (Polyakov et al., 2009). For each day of recording, we selected up to 200 events with the highest movement-onset velocities. Finally, all movement initiation events were visually inspected, and events with occlusions or false-positive movements were removed (17.8 ± 9.9% of events (mean ± SD)].

### ECoG–event synchronization and segmentation

We used time stamps accompanying clinical recordings to synchronize movement initiation events with ECoG recordings and generated 10 s ECoG segments centered around each event. ECoG segments with missing data and large artifacts, such as line noise, were removed by computing log-transformed spectral power density for each segment and discarding segments with power <0 dB or with abnormally high power at 115–125 Hz (>3 SDs) compared with all segments. With these bad ECoG segments removed, we computed log-transformed time–frequency spectral power using Morlet wavelets (Debnath and Shah, 2015). Power at each segment was then baseline subtracted, using a baseline defined as 1.5–1 s before each movement initiation event.

### Projecting power into regions of interest

Because electrode placement was clinically motivated and varied greatly across subjects, we projected the spectral power computed at every electrode into common regions of interest (ROIs) defined by the AAL (automated anatomical labeling) atlas (Tzourio-Mazoyer et al., 2002). Before projection, to combine all subjects, all right hemisphere electrode positions were flipped into the left hemisphere. Using EEGLAB and MATLAB, we mapped from electrodes to small, predefined brain regions by positioning a three-dimensional Gaussian (2 cm full-width at half-maximum) centered at each electrode position and calculating the value of the Gaussian at each small region (Delorme and Makeig, 2004; Bigdely-Shamlo et al., 2013; Peterson et al., 2018). The values across small regions were combined based on the AAL region boundaries, providing a mapping between each electrode and AAL region based on radial distance. We performed this projection procedure separately for each subject.

By summing the weights from these mappings across electrodes, we estimated the electrode density for each AAL region. We retained regions with an average electrode density >3 across subjects, resulting in the following eight ROIs: middle frontal, precentral, postcentral, inferior parietal, supramarginal, superior temporal, middle temporal, and inferior temporal (see Fig. 3). These eight ROIs represent where most of the electrodes were located across subjects. We then normalized the weights for each ROI so that they summed to 1. These normalized weights were used to perform a weighted average of electrode-level spectral power for every ECoG segment, generating a spectral power estimate at each region of interest.

After projecting single-event spectral power onto regions of interest, we computed the median value across events separately for each subject and region. We then averaged the event median spectral power across subjects to obtain group-level estimates for each region of interest. To mask spectral power patterns that were not significant, group-level spectral power for every frequency bin within each region of interest was then compared with a 2000-permutation bootstrap distribution generated from baseline time points. Nonsignificant differences from each bootstrap distribution were set to 0 (*p *>* *0.05, two-sided bootstrap statistics, false discovery rate correction; Benjamini and Hochberg, 1995).

### Single-event behavioral metadata features

We extracted multiple behavioral and environmental metadata features that quantify variations in movement parameters and environmental contexts (Donchin et al., 2002; Heldman et al., 2006; Ebner et al., 2009; Mooshagian et al., 2018). These features were later used as input variables for regression models of interevent spectral power and can be divided into four categories.

#### Timing features

Day of recording and time of day for each movement initiation event are used to capture long-term variations in the neural response.

#### Reach movement features

To quantify differences in the detected movements, we defined a reach as the maximum radial displacement of the wrist marker during the detected move state compared with its position at each movement initiation event. These features included the duration and magnitude of each reach. We also computed the 2D reach angle and transformed angles at 90–270^∘^ to range from 90^∘^ to −90^∘^, respectively. This transformation made the reach angle sensitive to vertical reach variations, with 90^∘^ for upward reaches and −90^∘^ for downward reaches. We also computed wrist marker radial speed during movement onset. Note that these movement features were based on the location of the video camera, which varied slightly across subjects and recording days.

#### Environmental feature

Based on results from the literature (Dumas et al., 2010; Derix et al., 2012); we were motivated to consider how environmental factors affect electrocortical power. Here, we examined the environmental factor of people talking during movement initiation. First, we cleaned the recorded audio signal using spectral noise gating (https://www.audacityteam.org), which performed 40 dB reduction on audio signal components that were similar to a selected noise period during rest. We then used the short-time Fourier transform to compute the spectral power from 370 to 900 Hz as a proxy for speech (Master et al., 2006). This power was divided by the total power at each time point, producing a ratio that is robust to broadband changes in the audio signal caused by noise. This speech ratio was smoothed using a first-order low-pass filter with 4.2 mHz cutoff to minimize the effects of transient changes in power because of noise. We then averaged this ratio from −1 to 1 s around each movement initiation event, generating a speech ratio feature that ranges from 0.0 to 1.0.

#### Bimanual reach features

While movement initiation event selection was based solely on contralateral wrist movement, the ipsilateral wrist can still move and may affect the electrocortical response. We quantified the relative magnitude of ipsilateral wrist movement by computing the ratio of the ipsilateral wrist reach magnitude to the sum of ipsilateral and contralateral reach magnitudes. In addition, we computed the temporal overlap between contralateral and ipsilateral move states over the duration of the entire contralateral wrist movement. Finally, we computed a binary feature that classified movements as either unimanual or bimanual based on the amount of temporal lag between contralateral and ipsilateral wrist movement onset. This feature was bimanual if a sequence of four consecutive move states of the ipsilateral wrist began either 1 s before contralateral wrist movement initiation or anytime during the contralateral wrist move state.

### Single-event spectral power linear regression

Using the 10 extracted behavioral features as independent variables, we fit a separate linear regression model to the spectral power at every electrode. While projecting onto cortical regions provided a useful visualization, we found that fitting regression models using projected power resulted in very poor model fits, likely because of electrodes with maximal power responses overlapping multiple regions and differing across subjects. All features were standardized before regression, with reach duration and reach magnitude features also being log transformed. We categorized the two timing features using one-hot encoding based on the day of recording and three 8 h segments (12:00 A.M. to 8:00 A.M., 8:00 A.M. to 4 P.M., and 4  P.M. to 12:00 A.M.) for time of day because we do not expect linear long-term power changes within and across days. For the dependent variable, we averaged spectral power over the first 0.5 s of movement onset, using previously validated low-frequency bands (LFBs; 8–32 Hz) and high-frequency bands (HFBs; 76–100 Hz; Miller et al., 2007). We then randomly selected 90% of each subject’s total contralateral arm movement events as training data, while withholding the remaining 10% for testing model generalizability. For each model, we independently pruned input features using forward selection, retaining features that improved the adjusted *R*^2^ for an ordinary least-squares fit. This procedure helped minimize overfitting because of too many independent variables.

For training, we applied a multiple linear regression model for event-by-event spectral power patterns (see Fig. 6*a*, scheme) defined as follows:

(1) $$y_{jkf}=\beta_{0kf}+\sum_{i=1}^{m}\beta_{ikf}x_{ij},$$

where *y_jkf_* is the spectral power for movement event *j* at electrode *k* averaged over frequency band *f*, during the first 0.5 s of movement initiation; *x_ij_* is feature *i* at event *j*; and *β_ikf_* is the coefficient for feature *i* at electrode *k* and frequency band *f* ($\beta_{0kf}$ is the intercept term). We minimized the Huber norm during model fitting to improve model robustness to outliers (Huber, 1964).

After training, we performed model validation by computing the *R*^2^ on withheld data, referred to as the “full model *R*^2^.” We also assessed the contribution of each behavioral feature independently by shuffling one feature, fitting a new model, and computing the *R*^2^ on the unshuffled, withheld data. This new *R*^2^ was subtracted from the full-model *R*^2^ to obtain $\Delta R^{2}$ as an estimate of that feature’s importance. We repeated this shuffling process and computation of $\Delta R^{2}$ across all model features.

We computed independent regression models using forward selection, along with *R*^2^ and $\Delta R^{2}$ scores, over all electrodes and for both low- and high-frequency bands. To minimize bias in our selection of training and testing data, we performed 200 random, independent train/test splits for every regression model, averaging the full-model *R*^2^, $\Delta R^{2}$, and coefficients across all splits. We balanced days of recording within each train and test set.

### Code accessibility

The code/software described in the article is freely available online at https://github.com/BruntonUWBio/naturalistic_arm_movements_ecog. For this study, we ran our code on a Z270 GAMING M7 (MS-7A57) machine running Ubuntu 18.04.4 LTS.

### Data availability

**Figure 2.**
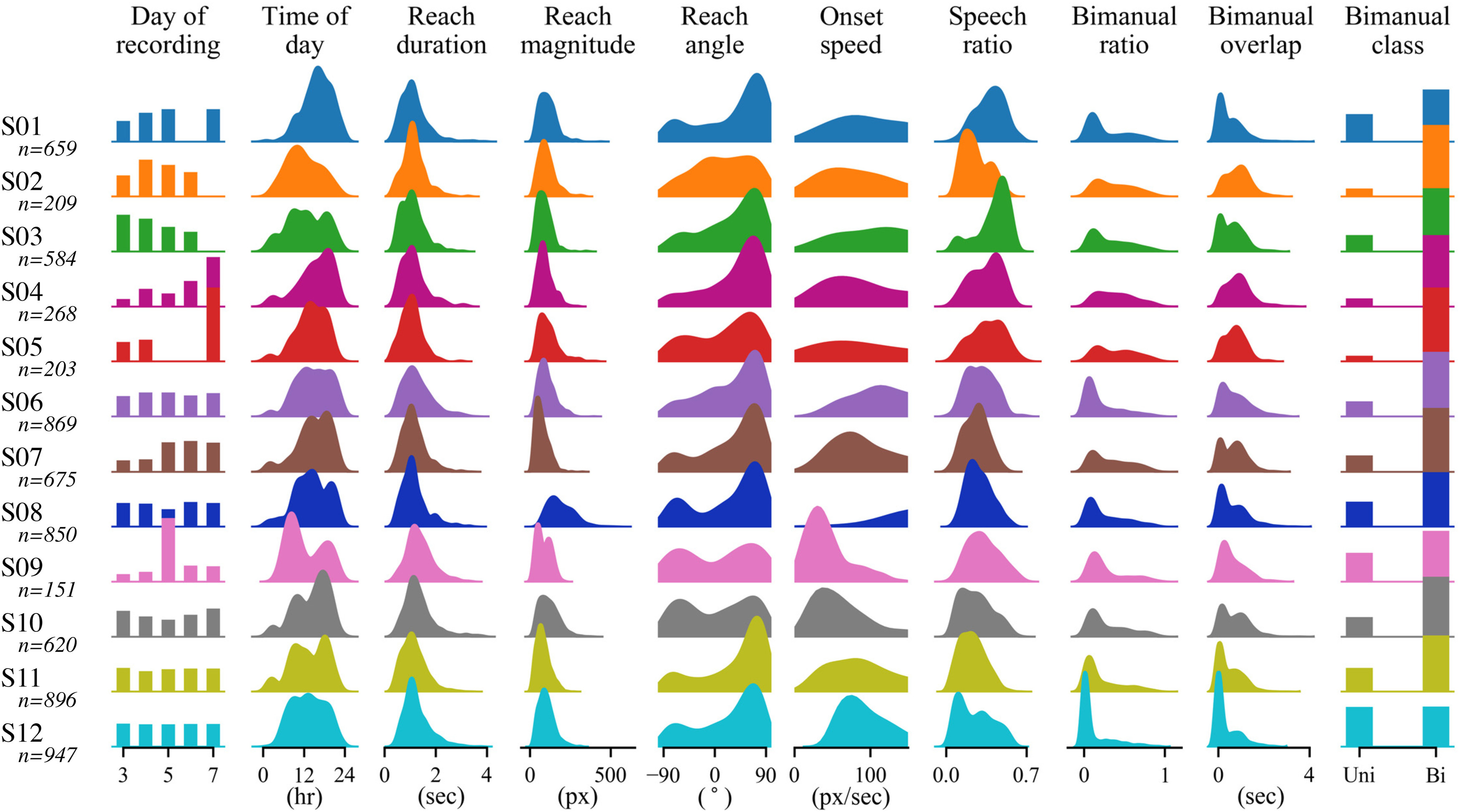
The distribution of extracted behavioral and environmental features show large intersubject variability. For each subject, features shown include timing (day of recording, time of day), reach parameters (duration, magnitude, angle, onset speed), environment (speech ratio), and bimanual factors (ratio, overlap, and class). All features significantly differed across subjects (Extended Data Figures 2-3, 2-4), reflecting the large variability among reach movements (Extended Data Figure 2-1). The total number of events for each subject was between 151 and 947 (median, 640 across subjects). Each distribution was normalized. These extracted features were used as inputs to the multiple regression models. Feature pairwise correlations are shown in Extended Data Figure 2-2. Note that 3 pixels = ∼1 cm.

**Figure 3.**
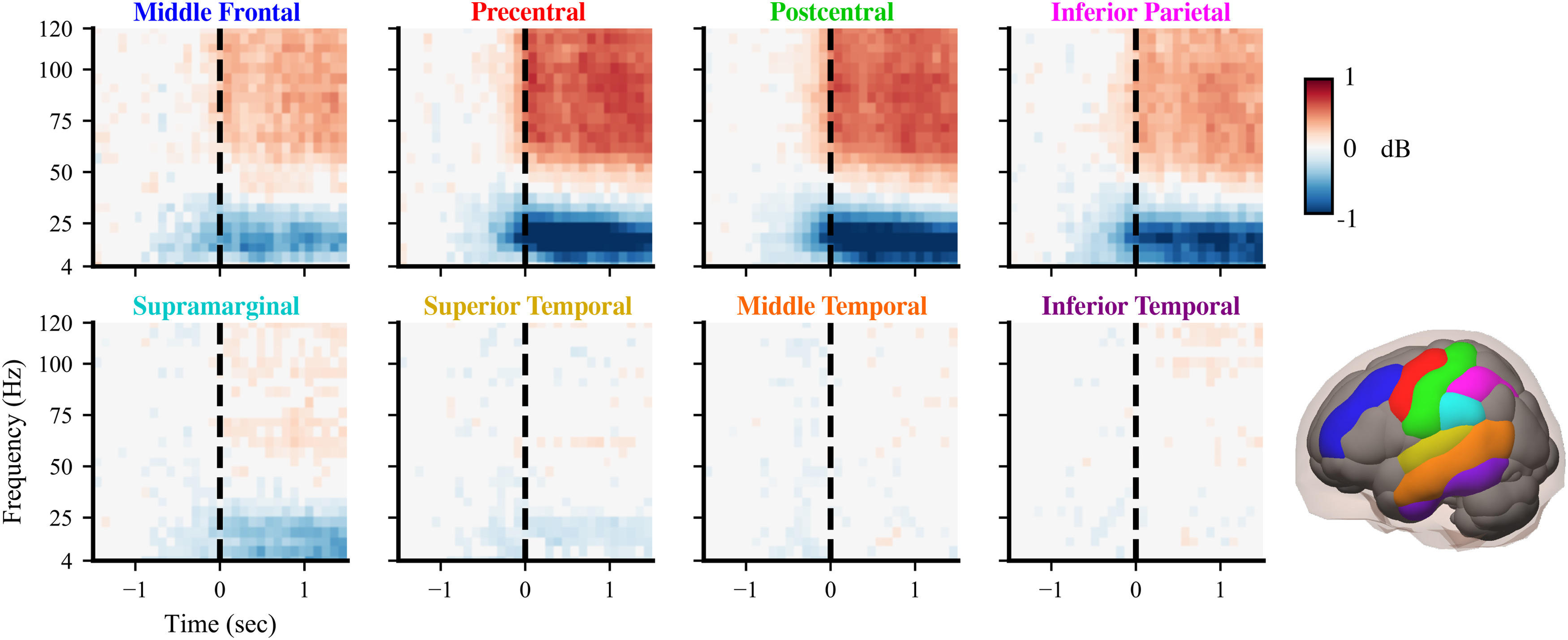
Group-level cortical spectral power changes are consistently localized to sensorimotor regions. Spectrograms show movement event-triggered spectral power patterns for eight cortical regions (highlighted in bottom right) summarized across all 12 subjects (see Extended Data Figure 3-1 for electrode-level spectral power). In general, low-frequency (4–30 Hz) power decreases and high-frequency (50–120 Hz) power increases at movement initiation (0 s), with the largest power fluctuations in frontoparietal sensorimotor areas. Spectral power was projected based on anatomic registration from electrodes onto the following eight regions of interest: middle frontal (blue), precentral (red), postcentral (green), inferior parietal (magenta), supramarginal (cyan), superior temporal (yellow), middle temporal (orange), and inferior temporal (purple). We subtracted the baseline power of the 1.5–1 s before movement initiation. Nonsignificant differences from baseline power were set to 0 (*p *>* *0.05).

**Figure 4.**
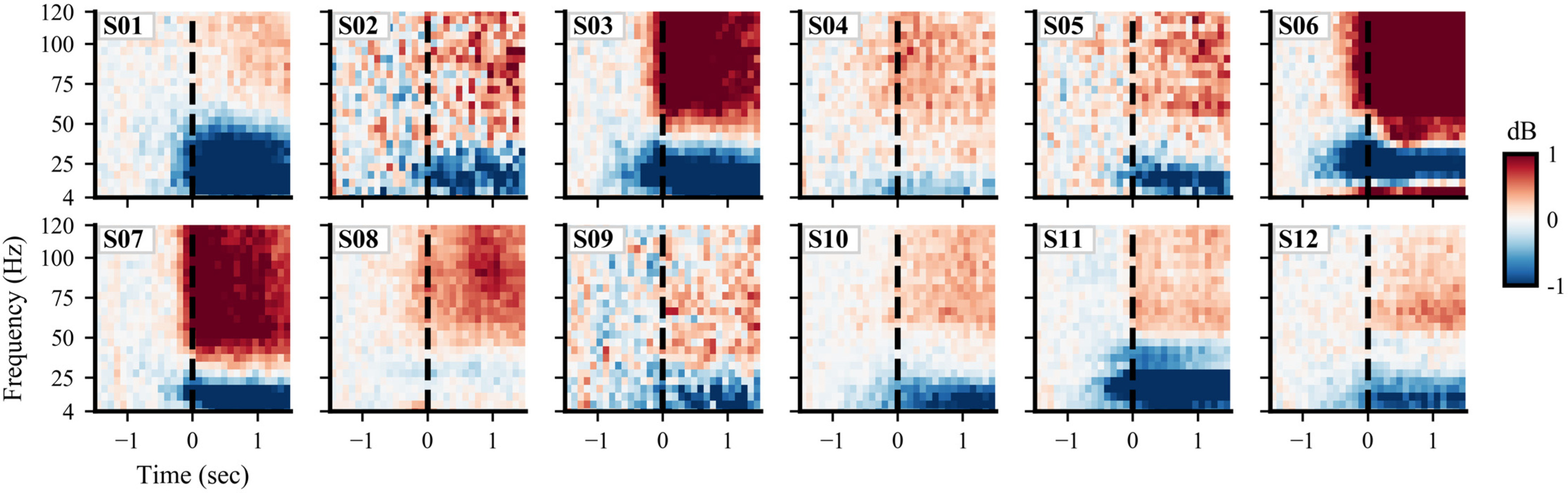
Spectral power patterns in the precentral region vary considerably across subjects. While some subjects show spectral power patterns similar to the group-level results in Figure 3, many deviate substantially from the group average pattern in both magnitude and frequency bands. The colormap indicates differences in spectral power relative to baseline 1.5–1 s before movement initiation (no statistical masking is used). For spectral power plots of the seven other regions of interest, see Extended Data Figures 4-1, 4-2, 4-3, 4-4, 4-5, 4-6, 4-7.

**Figure 5.**
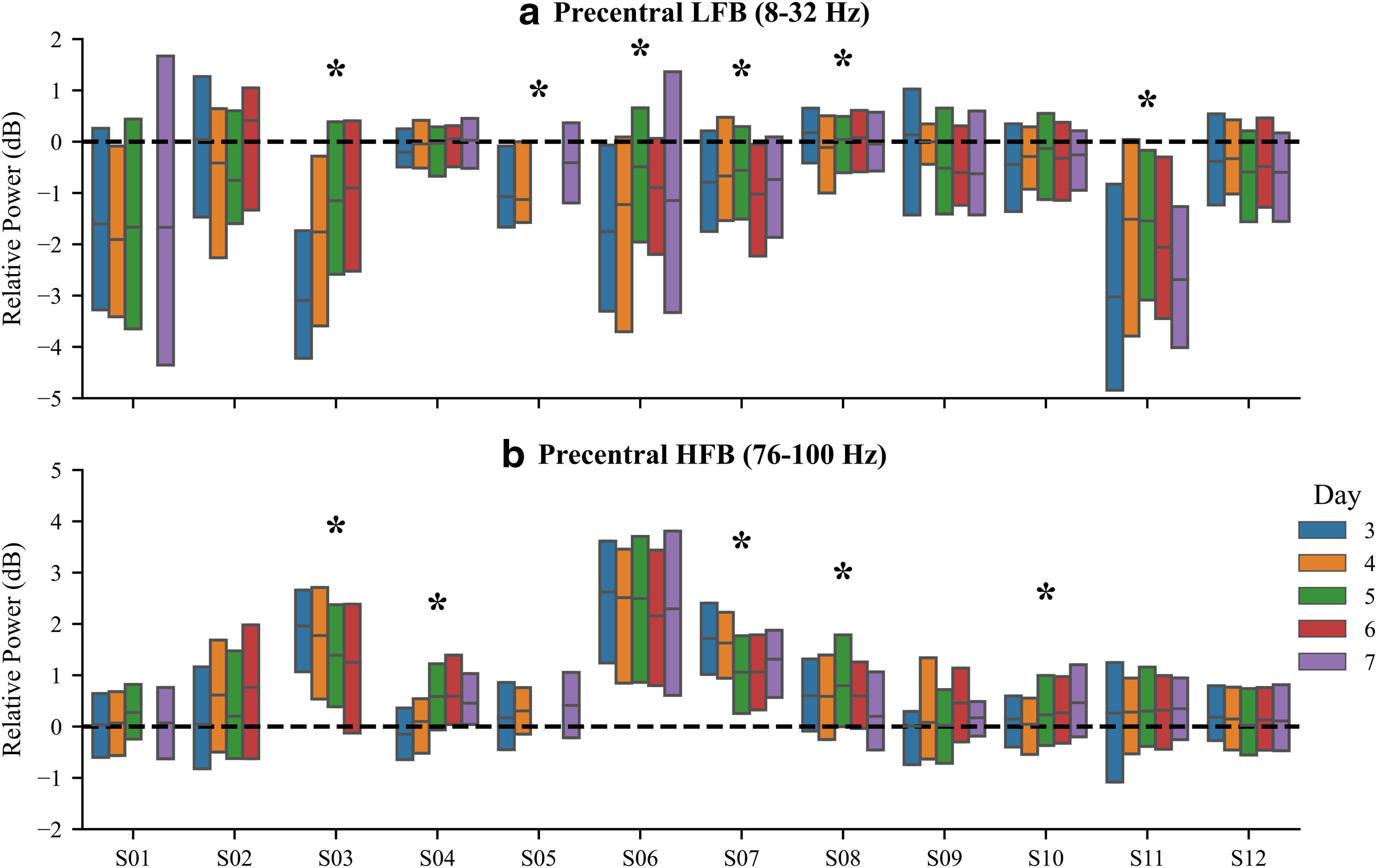
Precentral banded spectral power varies considerably across subjects and recording days. ***a***, ***b***, LFB (8–32 Hz; ***a***) and HFB (76–100 Hz; ***b***) spectral power in the precentral region was averaged over the first 0.5 s after movement onset. Boxplots show spectral power variability across events for every subject, separated by recording day. For each subject, a significant recording day effect on spectral power is denoted by an asterisk (*p* < 0.05, Kruskal–Wallis test). Despite the reduction in neural variability caused by baseline subtraction (Extended Data Figure 5-1), several subjects have significant recording day effects.

**Figure 6.**
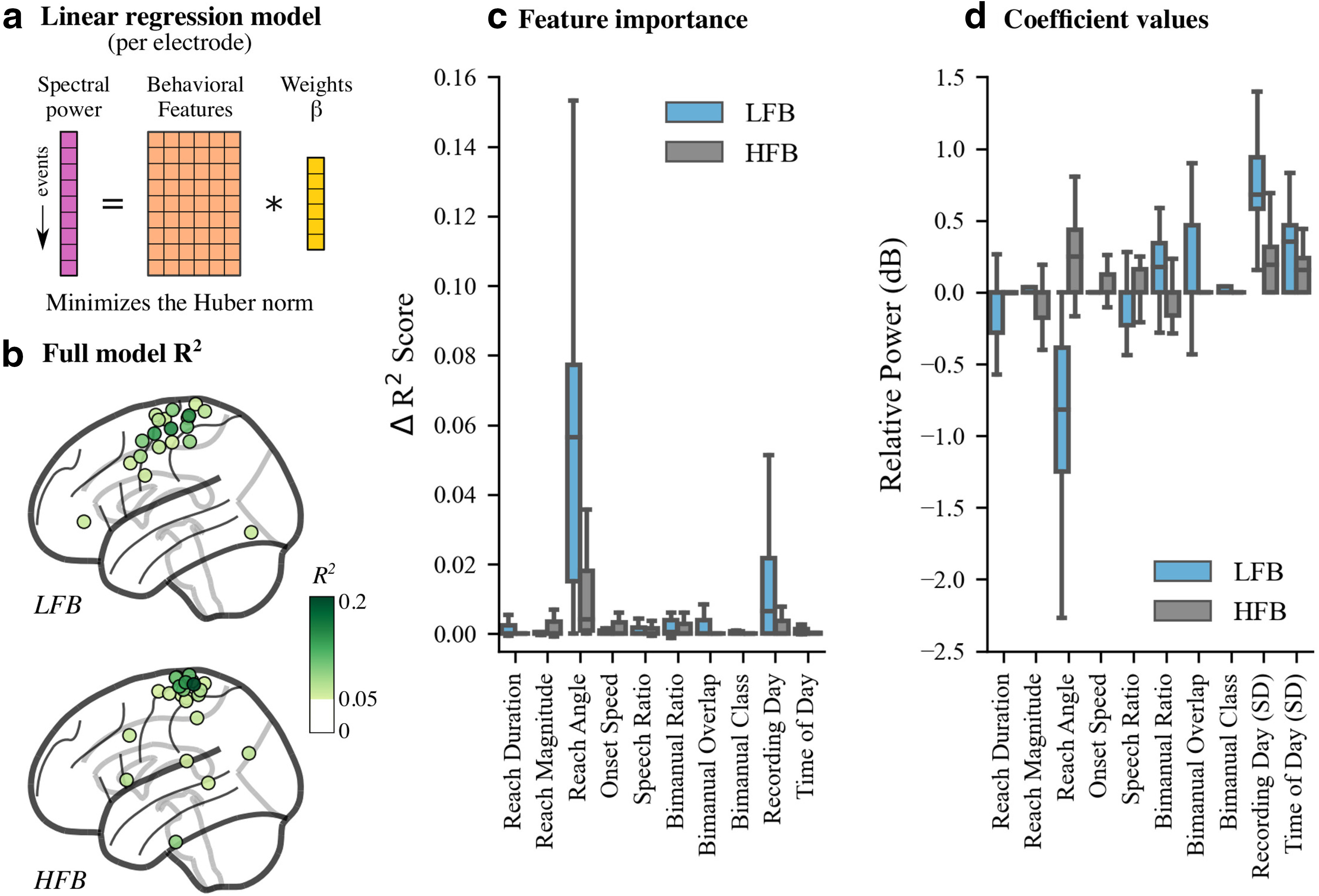
Event-by-event multiple regression models explain changes in neural spectral power using extracted behavioral and environmental features. ***a***, We fit multiple linear regression models at each electrode using behavioral features extracted from the videos (orange) and frequency-banded spectral power (magenta). Regression models minimized the Huber norm during model fitting to improve model robustness to outliers. ***b***, Models with the largest *R*^2^ scores on withheld data were primarily located in sensorimotor areas (see Extended Data Figure 6-1 for single subject *R*^2^ scores). ***c***, Reach angle and recording day were the most explanatory model features, especially when regressing low-frequency spectral power. Reach angle was also the most often retained feature in sensorimotor areas after forward selection (Extended Data Figure 6-2). ***d***, Regression coefficients indicate that upward reaches enhance the average spectral power pattern observed. Recording day and time of day both have large SDs across one-hot encoded variable coefficients, highlighting the effects of long-term temporal variability. Only models with $R^{2}>0.05$ on withheld data are shown for ***b–d***.

Our curated dataset is publicly available without restriction, other than citation, through Figshare at https://figshare.com/projects/Behavioral_and_neural_variability_of_naturalistic_arm_movements/78666. This public dataset contains synchronized neural and behavioral data that can be used to generate Figures 2-6.

10.1523/ENEURO.0007-21.2021.f2-1 Figure 2-1 Behavioral variability during naturalistic movements is large. ***a***, An example of tracked joint markers during 2 h of video monitoring for subject 01 (S01). Movement events of S01’s right wrist are visualized, as the subject’s electrodes were implanted in the left hemisphere. A heatmap of the locations of each joint is shown. ***b***, An example of 100 randomly selected movement trajectories of the right wrist for S01, shown as displacement in pixels from the rest position, demonstrates the large variability seen across naturalistic arm movements. The solid black line denotes median displacement across events. Events are aligned by time of movement initiation (vertical dashed line). ***c***, Because we selected movement initiation events of the wrist contralateral to the hemisphere with implanted electrodes, the median contralateral wrist displacements (orange lines) across all 12 subjects are substantially greater than ipsilateral wrist displacements (gray lines). Download Figure 2-1, EPS file.

10.1523/ENEURO.0007-21.2021.f2-2 Figure 2-2 Behavioral feature scatter matrix. Group-level feature covariance is assessed using a scatter matrix, with feature pair Pearson correlation coefficients overlaid. Reach magnitude is positively correlated with reach duration and onset speed. Reach duration is positively correlated with bimanual overlap because the maximum amount of possible overlap depends on reach duration. All three bimanual features are highly correlated to each other as well. Download Figure 2-2, EPS file.

10.1523/ENEURO.0007-21.2021.f2-3 Figure 2-3 Statistical table of the first five extracted behavioral and environmental features. Confidence intervals for day of recording, time of day, reach duration, reach magnitude, and reach angle are shown for each subject. The 95% confidence intervals were computed using bootstrap statistics with 5000 replicates. Download Figure 2-3, EPS file.

10.1523/ENEURO.0007-21.2021.f2-4 Figure 2-4 Statistical table of the last five extracted behavioral and environmental features. Confidence intervals for onset speed, speech ratio, bimanual ratio, bimanual overlap, and bimanual class are shown for each subject. The 95% confidence intervals were computed using bootstrap statistics with 5000 replicates. Download Figure 2-4, EPS file.

## Results

We describe behavioral and neural variability observed in multielectrode intracranial neural recordings and video from 12 human subjects during thousands of unstructured arm movements. Each subject had been implanted with ECoG electrodes for clinical monitoring, and we analyzed 3–5 d of simultaneously recorded video and electrophysiological data following surgery. We developed an automated and scalable approach to track upper-limb movements based on machine learning and then focused on analyzing spectral power changes associated with movements of the wrist contralateral to the hemisphere with implanted electrodes (Fig. 1*a–g*). ECoG monitoring was clinically motivated, so there was substantial variation in electrode placement among subjects (Fig. 1*h*). Because our focus was on arm-reaching behavior, we chose to analyze 12 subjects who were generally active during their monitoring and also had electrodes implanted over frontoparietal sensorimotor cortical areas.

### Behavior during naturalistic movements

The goal of our data processing pipeline was to automate both the identification of wrist movement initiation events and the description of behavioral and environmental features around each event. For each subject, we obtained simultaneously recorded neural activity and movement trajectories immediately before and after the initiation of each movement event (Fig. 1*e*). Briefly, two-dimensional wrist trajectories were estimated from the video recordings (Mathis et al., 2018) and then segmented into move or rest states. For simplicity of interpretation, we focused on movement initiation events of the wrist contralateral to the ECoG implantation hemisphere, detected during transitions from rest to move states. While we later analyzed ipsilateral wrist behavior to determine whether a contralateral wrist movement was bimanual, we did not use the ipsilateral wrist for detecting movement events.

The spontaneous wrist movement events that we identified include a wide variety of upper-limb movement behaviors. Because subjects were sitting in bed, a majority of the movements that we analyzed involved relatively little movement of the shoulders and elbows (Extended Data Fig. 2-1*a*). Most of the detected movements corresponded to actions such as reaching for a phone, eating, or touching one’s face. We confirmed that our event detection primarily identified contralateral wrist movements, as seen in Extended Data Fig. 2-1*c*.

To better assess behavior during wrist movement events, we obtained quantitative values of various movement features and associated environmental variables. We defined a reach as the maximum radial displacement of the wrist during the detected movement event, compared with the wrist position at movement initiation. We extracted 10 behavioral metadata features that quantified the time when each reach began, how the contralateral wrist moved during the reach, whether people were speaking during movement initiation, and how much both wrists moved during each movement.

We find that many metadata feature distributions show large within-subject and between-subject variations (Fig. 2). All 10 metadata features significantly differed across subjects (*p* < 0.001 for every feature, one-way Kruskal–Wallis test). See Extended Data Figs. 2-3 and 2-4 for further statistical details. The number of reaches detected across days of recording was fairly consistent, with the exceptions of subjects 04, 05, and 09, who each had 1 d representing most of the total events. As expected, detected movement events often occurred mostly during waking hours. Reach duration and reach magnitude show minimal intersubject variability, with most reaches lasting <2 s and covering <200 pixels (∼67 cm). For reach angle, the distributions tend to be bimodal, with peaks at ±90^∘^, indicating that detected events are biased toward upward and downward reaches, with few side-to-side reaches. Both onset speed and speech ratio distributions vary greatly across subjects, likely reflecting intersubject differences in the activities performed and the number of people visiting during the detected movement initiations. We also considered a number of features related to coordinated movements with the ipsilateral arm. For bimanual ratio and overlap features, the distributions are skewed toward unimanual movements of the contralateral limb, as expected from Extended Data Fig. 2-1*c*, with less skew for subjects 02, 04, and 05. In contrast, the bimanual class categorical feature is primarily skewed toward bimanual movements, indicating that the ipsilateral wrist is often moving, but only a small amount.

10.1523/ENEURO.0007-21.2021.f3-1 Figure 3-1 Spectral power across electrodes for each subject. Spectral power is shown averaged from 0 to 0.5 s across LFBs/HFBs. Colors reflect median values across events. Median values that were not significantly different from –1.5 to –1 s baseline were set to 0 (*p* > 0.05 with false discovery rate correction, bootstrap statistics using 2000 permutations). The far left column shows spectral power for all 12 subjects; only the electrodes with significant power are shown. Download Figure 3-1, EPS file.

10.1523/ENEURO.0007-21.2021.f4-1 Figure 4-1 Postcentral spectral power for each subject. Postcentral median spectral power across contralateral arm movement onset events is shown for each subject. Colors indicate the difference in spectral power relative to baseline (–1.5 to –1 s) in decibels. Movement onset is at time 0, denoted by dashed vertical line. No statistical masking is used. Download Figure 4-1, EPS file.

10.1523/ENEURO.0007-21.2021.f4-2 Figure 4-2 Middle frontal spectral power for each subject. Middle frontal median spectral power across contralateral arm movement-onset events is shown for each subject. Colors indicate the difference in spectral power relative to baseline (–1.5 to –1 s) in decibels. Movement onset is at time 0, denoted by dashed vertical line. No statistical masking is used. Download Figure 4-2, EPS file.

10.1523/ENEURO.0007-21.2021.f4-3 Figure 4-3 Inferior parietal spectral power for each subject. Inferior parietal median spectral power across contralateral arm movement onset events is shown for each subject. Colors indicate difference in spectral power relative to baseline (–1.5 to –1 s) in decibels. Movement onset is at time 0, denoted by dashed vertical line. No statistical masking is used. Download Figure 4-3, EPS file.

10.1523/ENEURO.0007-21.2021.f4-4 Figure 4-4 Supramarginal spectral power for each subject. Supramarginal median spectral power across contralateral arm movement-onset events is shown for each subject. Colors indicate the difference in spectral power relative to baseline (–1.5 to –1 s) in decibels. Movement onset is at time 0, denoted by dashed vertical line. No statistical masking is used. Download Figure 4-4, EPS file.

10.1523/ENEURO.0007-21.2021.f4-5 Figure 4-5 Superior temporal spectral power for each subject. Superior temporal median spectral power across contralateral arm movement-onset events is shown for each subject. Colors indicate the difference in spectral power relative to baseline (–1.5 to –1 s) in decibels. Movement onset is at time 0, denoted by dashed vertical line. No statistical masking is used. Download Figure 4-5, EPS file.

10.1523/ENEURO.0007-21.2021.f4-6 Figure 4-6 Middle temporal spectral power for each subject. Middle temporal median spectral power across contralateral arm movement-onset events is shown for each subject. Colors indicate the difference in spectral power relative to baseline (–1.5 to –1 s) in decibels. Movement onset is at time 0, denoted by dashed vertical line. No statistical masking is used. Download Figure 4-6, EPS file.

10.1523/ENEURO.0007-21.2021.f4-7 Figure 4-7 Inferior temporal spectral power for each subject. Inferior temporal median spectral power across contralateral arm movement-onset events is shown for each subject. Colors indicate the difference in spectral power relative to baseline (–1.5 to –1 s) in decibels. Movement onset is at time 0, denoted by dashed vertical line. No statistical masking is used. Download Figure 4-7, EPS file.

We also assessed group-level correlations between feature pairs, finding high correlations for three reach parameter feature pairs and between all three bimanual feature pairs (Extended Data Fig. 2-2). Reach magnitude positively correlates with reach duration (*r *=* *0.26) and onset speed (*r *=* *0.56), meaning that reaches tended to cover more distance when they lasted longer or had higher onset speed. Reach duration is also positively correlated with bimanual overlap (*r *=* *0.48) because of movements with long durations having more possible overlap time. The high correlations between bimanual features (pairwise Pearson correlation coefficients: overlap vs ratio, *r *=* *0.51; class vs ratio, *r *=* *0.50; and overlap vs class, *r *=* *0.61) indicates that contralateral wrist movements classified as bimanual generally show increased overlap between ipsilateral and contralateral movements and increased ipsilateral amplitude relative to contralateral, as expected.

### Intracortical spectral power during naturalistic movements

We find a consistent set of group-level spectral power patterns, largely localized in frontoparietal sensorimotor cortical regions. After aligning curated wrist movement events with preprocessed ECoG recordings, we computed time–frequency spectral power at each electrode and then visualized group-level spectral patterns projected onto common regions of interest for all subjects. Generally, we find the expected pattern of low-frequency (∼4–30 Hz) spectral power decrease and high-frequency (∼50–120 Hz) power increases during movement initiation across multiple cortices (Fig. 3) similar to previous findings during controlled movement experiments (Miller et al., 2007). Because ECoG electrode placement varied across subjects, we visualized group-level neural activity by projecting power at every electrode onto the following eight common cortical regions of interest (Bigdely-Shamlo et al., 2013): middle frontal, precentral, postcentral, inferior parietal, supramarginal, superior temporal, middle temporal, and inferior temporal. Maximal power deviations primarily occur near movement onset, as expected. Spectral power deviations are largest in magnitude in precentral, postcentral, and inferior parietal regions, which are located in sensorimotor areas of the brain. The middle frontal region also contains strong power fluctuations that could indicate motor planning and possible recruitment of the supplementary motor area. In addition, low-frequency power decreases appear more spatially widespread than high-frequency power increases and are also present in supramarginal and superior temporal regions. As expected, all three temporal cortical regions contain minimal movement-related spectral power fluctuations.

Despite consistent group-level spectral power patterns across cortical regions, we identified considerable spectral power variability across subjects (Extended Data Fig. 3-1). For instance, the precentral region shows the same low/high-frequency power pattern for each subject (Fig. 4), but the amplitudes and frequency bands of maximal power deviation differ widely across subjects. Subjects 03, 06, 07, 08, and 11 show increased power at high frequencies up to 120 Hz, while subjects 09 and 12 have increased power primarily between 60 and 80 Hz. For subjects 04 and 08, low-frequency power decreases occur across narrower frequency bands compared with the other subjects. In addition to arising from intersubject differences in neural anatomy and connectivity, these spectral power variations may reflect variability in daily activities, electrode placement, medication, and seizure foci among subjects (Struck et al., 2015; Skarpaas et al., 2018). Spectral power plots for the seven other regions of interest are shown in Extended Data Figs. 4-1, 4-2, 4-3, 4-4, 4-5, 4-6, 4-7.

In addition to intersubject neural variability, we also identified notable changes in movement-related neural activity across recording days for several subjects (Fig. 5). We analyzed spectral power in the precentral region averaged over the 0.5 s following movement onset and split into LFBs (8–32 Hz) and HFBs (76–100 Hz) bands, similar to previous research (Miller et al., 2007). Statistical significance for each subject was computed by pooling over all electrodes for that subject (Extended Data Fig. 1-1). For both frequency bands, spectral power significantly differed across subjects (*p* < 0.001 for both bands, one-way Kruskal–Wallis test). For further statistical details, see Table 1. We also found a significant effect of recording day in LFBs for subjects 03 (*p* < 0.001, Kruskal–Wallis test), 05 (*p* = 0.013), 06 (*p* < 0.001), 07 (*p* = 0.041), 08 (*p* = 0.013), and 11 (*p* < 0.001), as well as in HFBs for subjects 03 (*p* = 0.002), 04 (*p* < 0.001), 07 (*p* < 0.001), 08 (*p* = 0.004), and 10 (*p* = 0.006). For further statistical details, see Table 2. Surprisingly, these significant recording day effects appear for several subjects despite baseline-subtracting spectral power features. Yet, baseline subtraction does substantially reduce neural variability across recording days (Extended Data Fig. 5-1), as expected.

**Table 1:** Statistical table for group-level spectrograms and precentral banded spectral power

Measure	Data structure	Type of test	95% confidence interval
Group-level spectrograms (Fig. 3)	Non-normal	Bootstrap statistics	
Precentral banded spectral power across subjects (Fig. 5)	Non-normal	One-way Kruskal– Wallis test	S01 (LFB, −1.87 to −1.29; HFB, 0.03–0.23)
			S02 (LFB, −1.12 to −0.31; HFB, 0.07–0.64);
			S03 (LFB, −2.11 to −1.73; HFB, 1.39–1.67)
			S04 (LFB, −0.17 to 0.01; HFB, 0.32–0.52)
			S05 (LFB, −0.7 to −0.38; HFB, 0.2–0.48)
			S06 (LFB, −1.63 to −1.11; HFB, 2.02–2.37)
			S07 (LFB, −0.84 to −0.51; HFB, 1.14–1.34)
			S08 (LFB, −0.3 to 0.04; HFB, 0.36–0.63)
			S09 (LFB, −0.57 to −0.08; HFB, −0.11 to 0.23)
			S10 (LFB, −0.87 to −0.35; HFB, 0.01–0.3);
			S11 (LFB, −2.3 to −1.86; HFB, 0.09–0.34);
			S12 (LFB, −0.66 to −0.44; HFB, 0.06–0.2)

Confidence intervals for LFB (8–32 Hz) and HFB (76–100 Hz) spectral power are shown for each subject. The 95% confidence intervals were computed using bootstrap statistics with 5000 replicates. We did not include confidence intervals for group-level spectrograms because of the high number of comparisons performed. S, Subject.

**Table 2:** Statistical table for precentral banded spectral power, separated by recording day

Measure	Data structure	Type of test	95% confidence interval
Precentral banded spectral power across recording days (Fig. 5, LFB)	Non-normal	One-way Kruskal– Wallis test	S01 (day 3, −2.07 to −0.9; day 4, −2.77 to −1.52
			Day 5, −1.96 to −1.04; day 7, −1.8 to −0.66)
			S02 (day 3, −0.62–0.6; day 4, −1.61 to −0.38;
			day 5, −2.67 to −0.32; day 6, −0.72 to 0.39)
			S03 (day 3, −3.12 to −2.46; day 4, −2.29 to −1.58;
			day 5, −1.55 to −0.86; day 6, −1.71 to −0.77)
			S04 (day 3, −0.51 to 0.11; day 4, −0.3 to 0.24; day 5,
			−0.51 to 0.07; day 6, −0.31 to 0.07; day 7, −0.16 to 0.11)
			S05 (day 3, −1.22 to −0.44; day 4, −1.23 to −0.48;
			day 7, −0.56 to −0.18);
			S06 (day 3, −2.26 to −1.42; day 4, −2.58 to −1.61; day 5,
			−1.1 to −0.4; day 6, −2.08 to −0.94; day 7, −1.65 to 0.18)
			S07 (day 3, −1.12 to −0.38; day 4, −0.73 to −0.02; day 5,
			−0.84 to −0.42; day 6, −1.24 to −0.8; day 7, −0.93 to 0.09)
			S08 (day 3, −0.72 to 0.55; day 4, −1.06 to −0.18; day 5,
			−0.23 to 0.09; day 6, −0.18 to 0.11; day 7, −0.09 to 0.18)
			S09 (day 3, −1.42 to 0.6; day 4, −0.55 to 0.32; day 5,
			−0.65 to −0.01; day 6, −0.99 to 0.51; day 7, −1.14 to 0.29)
			S10 (day 3, −1.3 to 0.0; day 4, −0.56 to −0.13; day 5,
			−0.59 to −0.09; day 6, −2.03 to −0.37; day 7, −1.22 to −0.27)
			S11 (day 3, −3.2 to −2.24; day 4, −2.18 to −1.31; day 5,
			−1.91 to −1.28; day 6, −2.18 to −1.51; day 7, −3.06 to −1.68)
			S12 (day 3, −0.58 to −0.2; day 4, −1.02 to −0.21; day 5,
			−0.86 to −0.39; day 6, −0.72 to −0.32; day 7, −0.85 to −0.47)
			
Precentral banded spectral power across recording days (Fig. 5, HFB)	Non-normal	One-way Kruskal– Wallis test	S01 (day 3, −0.11 to 0.34; day 4, −0.36 to 0.17; day 5, 0.17–0.55; day 7, −0.06 to 0.21)
			S02 (day 3, −0.35 to 0.66; day 4, 0.08–0.98;
			day 5, −0.72 to 0.76; day 6, 0.06–1.02)
			S03 (day 3, 1.6–2.0; day 4, 1.44–1.91;
			day 5, 1.16–1.64; day 6, 0.41–1.41)
			S04 (day 3, −0.45 to 0.12; day 4, −0.23 to 0.17; day 5,
			0.35–0.88; day 6, 0.49–0.93; day 7, 0.32–0.62)
			S05 (day 3, −0.12 to 0.5; day 4, 0.03–0.58;
			day 7, 0.2–0.58)
			S06 (day 3, 2.08–2.71; day 4, 1.76–2.41; day 5,
			1.96–2.58; day 6, 1.39–2.33; day 7, 1.71–2.83)
			S07 (day 3, 1.45–1.9; day 4, 1.23–1.76; day 5,
			0.9–1.26; day 6, 0.82–1.13; day 7, 1.15–1.68)
			S08 (day 3, −0.11 to 0.88; day 4, 0.05–0.72; day 5,
			0.6–1.05; day 6, 0.4–0.71; day 7, 0.13–0.47);
			S09 (day 3, −0.93 to 0.32; day 4, −0.52 to 1.04; day 5,
			−0.24 to 0.21; day 6, −0.02 to 0.73; day 7, −0.3 to 0.4)
			S10 (day 3, −0.33 to 0.28; day 4, −0.14 to 0.23; day 5,
			0.08–0.47; day 6, −0.37 to 0.55; day 7, −0.04 to 0.57)
			S11 (day 3, −0.24 to 0.36; day 4, −0.26 to 0.34; day 5,
			0.16–0.52; day 6, 0.04–0.4; day 7, 0.02–0.81)
			S12 (day 3, 0.08–0.34; day 4, −0.26 to 0.23; day 5,
			−0.05 to 0.26; day 6, 0.01–0.26; day 7, 0.04–0.34)

Confidence intervals for LFB (8–32 Hz) and HFB (76–100 Hz) spectral power are shown for each subject, separated by recording day. The 95% confidence intervals were computed using bootstrap statistics with 5000 replicates. S, Subject.

10.1523/ENEURO.0007-21.2021.f5-1 Figure 5-1 Baseline subtraction minimizes the spectral power variability across recording days. The SD of average low-frequency band (8–32 Hz) and high frequency band (76–100 Hz) spectral power across recording days is shown with and without subtraction of –1.5 to –1 s baseline values. Baseline subtraction reduces the variation across recording days, as expected, but clear variability remains. Download Figure 5-1, EPS file.

### Modeling single-event spectral power with behavioral features

We developed a robust multiple variable linear regression model to explain single-event spectral power at each intracranial electrode using our 10 behavioral metadata features (Fig. 6*a*). For each electrode, we modeled LFB and HFB spectral power averaged over the 0.5 s following movement onset (Fig. 1*i*). For every model, behavioral features were pruned independently using forward selection to avoid overfitting. We assessed model performance on randomly withheld movement events by computing an *R*^2^ score (referred to as the full model *R*^2^). To assess the contributions of each individual feature, we shuffled the training labels of that feature, fit a new linear model, and subtracted the *R*^2^ values on withheld data from the full model *R*^2^ values of this model to obtain an estimate of feature importance. Higher $\Delta R^{2}$ values indicate features that explain more variance.

10.1523/ENEURO.0007-21.2021.f6-1 Figure 6-1 Single-subject regression model fits. Full model *R*^2^ scores on withheld data are shown across subjects for LFB/HFB spectral power features. The far right column displays electrodes across all subjects with *R*^2^ > 0 averaged across 200 train/test splits. Well fit models with the largest positive *R*^2^ values are consistently located in frontoparietal sensorimotor areas for most subjects. Download Figure 6-1, EPS file.

10.1523/ENEURO.0007-21.2021.f6-2 Figure 6-2 Reach angle is the most consistently retained feature for regression models in sensorimotor areas. The average probability of retaining each feature across subjects following forward selection feature pruning is shown, projected to the eight regions of interest (SD is in parentheses). Regression models were fit to single-event LFB or HFB spectral power features. Reach angle is the feature most often retained, followed by reach duration, reach magnitude, bimanual ratio, and bimanual overlap. Download Figure 6-2, EPS file.

For both frequency bands, intracortical activity variability is best explained by the fit of our models to electrodes located in frontoparietal sensorimotor areas (Fig. 6*b*, Extended Data Fig. 6-1). This finding matches well with the spatial distribution of spectral power (Fig. 3). However, all *R*^2^ scores are at most 0.25, indicating that even the best models cannot explain >75% of the variance in the withheld data. Among individual features, we find that reach angle and day of recording are the most informative (Fig. 6*c*). Reach angle is also the most often retained feature following forward selection in sensorimotor regions (Extended Data Fig. 6-2), indicating its importance for modeling neural activity during movement onset. In addition, both reach angle and day of recording have the largest coefficient magnitudes among behavioral features in the regression models (Fig. 6*c*). The coefficients for reach angle indicate that upward reaches are associated with decreased low-frequency power and increased high-frequency power compared with the average response. In other words, upward reaches tend to increase the magnitude of the spectral power pattern seen. The SD of the coefficients corresponding to recording day are surprisingly large, indicating that baseline-subtracted neural responses still vary across long time scales usually not captured in short, controlled experiments. This observation highlights the importance of properly accounting for long-term temporal effects when understanding and decoding neural recordings.

## Discussion

Our results demonstrate that electrocortical correlates of naturalistic arm movements in humans corroborate findings from controlled experiments on average, as we had hypothesized. However, we found high behavioral and neural variability during naturalistic movements across participants and recording days. Using multiple regression modeling, we were able to partially explain this event-by-event electrocortical variability using behavioral metadata features extracted from video recordings. In general, we find that results from controlled upper-limb reaching tasks do generalize to naturalistic movements on average, but naturalistic movements involve considerable event-by-event neural variability that cannot be fully explained by simple behavioral and environmental measures.

Across subjects, we observe an average decrease in low-frequency band cortical power and an increase in high-frequency band cortical power during naturalistic upper-limb movement initiation, consistent with previous controlled studies (Miller et al., 2007; Gabriel et al., 2019). Decreases in low-frequency power are thought to reflect changes in the current neural state if a new or unexpected event occurs (Engel and Fries, 2010). In our study, the neural state can be disrupted during movement initiation by a variety of factors, such as increased attention or prediction error once the arm is in motion. In contrast, high-frequency power increases may indicate active sensorimotor processing (Başar et al., 2001; Manning et al., 2009; Tam et al., 2019; Branco et al., 2019a,b). Low-frequency and high-frequency power changes are thought to represent two separate processes (Miller et al., 2009; van Kerkoerle et al., 2014), which could explain the difference seen in the spatial spread of cortical power changes between low and high frequencies. In our study, the frequency bands of the maximum spectral power responses do differ across subjects, suggesting that the processes underlying the low- and high-frequency bands vary across subjects. This intersubject variability reinforces the importance of assessing both subject-specific neural responses and group-level activity.

Despite showing the expected cortical pattern on average, naturalistic reaches, exhibited notable behavioral and neural variability across subjects and recording days. This high variability may reflect variations in sensory input and movement constraints because of different types of behaviors (Lisberger and Medina, 2015). Categorizing such behaviors during reaching would be challenging, however, because of many possible neurally relevant behavior types and a lack of objective measures that can properly discriminate such behaviors without user-defined labels (Gomez-Marin et al., 2014). The neural variation seen across recording days could be caused by several factors, including changes in medication, seizure frequency, and alertness while recovering from ECoG implantation surgery. Similar long-term, interday variability has been observed in previous EEG and ECoG studies (Melnik et al., 2017; Gliske et al., 2018; Nurse et al., 2018). It is also worth noting that these day-to-day changes in ECoG spectral power are small in magnitude (±1–2 dB) relative to spectral power without any baseline values subtracted (∼10–50 dB). Furthermore, recent research suggests that despite long-term neural recording variability, low-dimensional representations of this activity remain stable over long periods of time (Gallego et al., 2020).

During regression modeling, we find that our models only explained at most 25% of the variability; this measure is low, but not unusual given the single-event noise in the electrocortical signal (Liang and Bougrain, 2012). Furthermore, some of this variability may be explained by other movement behaviors beyond what we quantified using our pose-tracking methodology (Musall et al., 2019). These low scores may also reflect the simplicity of our linear models. While studies have shown evidence of nonlinear relationships between electrocortical activity and behavior (David et al., 2004; Ting and McKay, 2007), we chose linear regression models because they provide easily interpretable results and allow straightforward assessments of individual feature contributions.

Our regression model identified vertical reach angle and day of recording as the most explanatory features. The importance of vertical reach angle makes sense because upward reaches require more effort and activate different muscles than downward reaches. In addition, population neural activity has been shown to robustly encode reach direction (Georgopoulos et al., 1986; Hu et al., 2018). We did not include a reach angle feature sensitive to horizontal movements because reach angle distributions were skewed toward vertical angles at ±90^∘^, as seen in Figure 2. The day of the recording feature was also found to explain some of the neural variance captured by regression modeling. This finding is sensible given the significant interday neural variability seen for several subjects.

Our study has several important limitations. Because we are performing human ECoG research, we are studying subjects who have epilepsy and are recovering from electrode implantation surgery, which may introduce confounding effects because of medication and seizure location. To address this issue, we ignored data from the first 2 d postsurgery, removed electrodes with abnormal activity, and assessed movements across multiple days to avoid single-day bias. In addition, we assessed behavior from subjects who were confined to a hospital bed and selected parabolic movement trajectories for further analysis. Thus, these movements were not 100% naturalistic, but the arm movements we analyzed were still spontaneous and unconstrained by experimental factors. Another limitation is that the clinical video monitoring system includes only one camera, whose view can be obstructed by people and various objects throughout the day. We minimized obstruction effects by selecting movement events with high confidence scores in the event detection algorithm and by manually reviewing all detected events to check whether they were actual movements and not false positives, but using multiple cameras would extend body tracking to 3D in future studies. Additionally, the video camera was positioned by the clinical staff and was vertically rotated away by the clinical staff during private times, meaning that the video view changed slightly throughout the day. We minimized the effect of such rotations on our behavioral features by excluding camera rotation events and using movement features that were relative to the start of each reach.

Our results underline the importance of studying naturalistic movements and understanding neural variability across multiple days. Our approach leverages pre-existing clinical setups and could be extended to other movements and behaviors, such as grasping objects, sleep/wake transitions, and conversing with others. Future setups with multiple RGB or depth cameras would allow for improved motion-tracking performance and the ability to analyze more complex 3D trajectories (Desmarais et al., 2020; Mathis et al., 2020). In addition, our approach could be extended beyond epilepsy monitoring to study neural correlates of naturalistic movements during long-term scalp EEG recordings or chronic neural implants in other clinical cohorts (Duun-Henriksen et al., 2020; Gilron et al., 2020). It is also worth noting that such markerless pose tracking has also been used to track lower-limb movements (Chambers et al., 2019; Cao et al., 2021), provided the subject remains within view of the cameras.

More broadly, our results have implications for developing novel brain–computer interfaces that can decode neural data across subjects in natural environments. For instance, movement data from many subjects could be combined to train decoders that generalize to new subjects with minimal retraining and are robust to a richer set of behavioral and environmental contexts. By publicly releasing our curated dataset, we hope to spur further research that enhances our understanding of naturalistic behavior and informs the development of next-generation brain–computer interfaces.

## Synthesis

Reviewing Editor: Mark Laubach, American University Decisions are customarily a result of the Reviewing Editor and the peer reviewers coming together and discussing their recommendations until a consensus is reached. When revisions are invited, a fact-based synthesis statement explaining their decision and outlining what is needed to prepare a revision will be listed below. The following reviewer(s) agreed to reveal their identity: NONE.

Both reviewers identified some key issues with your manuscript. Please address all points that are noted below.

Reviewer 1

Here, authors took an innovative approach to characterize behavior and neural activity of naturalistic arm movements in people with epilepsy. Their rationale was that this approach is feasible when ECoG is used with conjunction of motion capturing. Their results strengthen the notion that this analysis is feasible and may shed light towards the neural and motor control of unstructured and spontaneous arm movements. Furthermore, their results stress out the importance of looking the inter-subject and across events variability, which was used to model certain features. Among all features, only two strongly explained this variability. Taken together with this innovative approach, the findings of the present study can be used to test similar tasks in other clinical cohorts.

1) Authors should revise the text for typos etc (ex. Line 376: “cold” should be “could”).

2) Authors should add a section about future directions/clinical implications. In this section they can talk what the next steps could be and how this approach can be possibly used in other clinical cohorts. Also, authors should discuss whether this approach can be used to test naturalistic movements with lower extremity.

3) In the limitation section, authors should acknowledge the fact that the behavior was assessed while they were confined to a hospital bed. Therefore, these actions are not 100% naturalistic, but the arm movements are spontaneous and not constrained by experimental factors (e.g., movement time, target size and location).

4) Similar to the comment above, authors should consider revising the title. The word “naturalistic” may not be appropriate or accurate. They should consider replacing this word with “spontaneous”.

Reviewer 2

I believe that the manuscript addresses two of the major problems of the study of motor control: the lack of natural conditions and the necessity of providing movement correlates in humans. Towards this end, the authors perform a careful study addressing two of the hard points in these kinds of problems: identifying relevant features both of behavior and of neural data.

The manuscript addresses the considerable challenge of explaining the neural basis of movement by analyzing ECoG recordings during naturalistic movement performance. Unlike most common experiments where the results are performed in the controlled conditions of numerous repetitions of the same movement under the same precise conditions, here the authors carried out a series of experiments aimed at recording and analyzing thousands of natural arm movements, together with ECoG recordings from epilepsy patients.

I believe that the manuscript addresses two of the major problems of the study of motor control: the lack of natural conditions and the necessity of providing movement correlates in humans. Towards this end, the authors perform a careful study addressing two of the hard points in these kinds of problems: identifying relevant features both of behavior and of neural data.

One of the major challenges of analyzing unconstrained movements is their intrinsic variability. Amplitudes, orientations and targets are not defined, which turns movement characterization into a formidable task.

The authors characterized thousands of spontaneous movements enabling scalable analysis of video that was acquired simultaneously with the intracranial recordings. We characterized the variability of both naturalistic upper-limb reaching movements and the corresponding changes in cortical spectral power.

Their hypothesis is that naturalistic reaches would be associated with transient low-frequency power decreases and high-frequency power increases, localized from fronto-parietal sources. They also assessed the variability of these event-related spectral power fluctuations and performed regression to partially explain this neural activity using behavioral frames.

Issues

I think their approach is courageous. However, my major concern is that the manner in which they select movement related features is unprincipled. In particular, movements were characterized not with a motion tracking system but rather from video annotation, which has to be done manually. A justification of the kinds of motor features you used should be provided.

The features were extracted ad hoc, to quantify variations in movement parameters and environmental contexts. These features were later used as input variables for regression models of inter-event spectral power and can be divided into 4 categories: Timing, Reach movement, Environmental, bimanual reach features.

- Wrist movements were labelled manually, but some criteria to select movement events were clearly ad hoc. Why can wrist movements only be parabollic? If the target is to analyze naturalistic movements, imposing this non-trivial constraint seems to defeat the purpose.

- With regard to the ECoG signals (I am agnostic as to this kind of signal), but why do you only use electrode power alone. Furthermore, you do not specifically explain how did you cope with inter-subject variability in so far electrodes are placed differently.

- The reach movement feature extraction seems pretty much ad hoc. How did you correct for focal differences between images of different days / subjects?

- The section of environmental features is ambiguously described. However, one possible method of validation is correlation or a metric of similarity between htem.

- The variability across subjects is within norm, but I would expect you to validate the specific features by statistically comparing across subjects.

- What specific criterion did you use to organize your frequency separation?

- You need to explain better the kinds of models you use for single-event spectral power with behavioural features (expand on FIG 6a). Is it just a linear model?

Minor Issues

- The presentation of events is very hard to read (FIG5)

## Author Response

We are grateful to both reviewers for their feedback and thoughtful suggestions. Based on these reviews, we have made substantive changes to our manuscript and believe that the revisions have improved the quality and clarity of our manuscript. All changes are highlighted in our tracked changes document, with additions in blue and deletions in red. In addition, we have responded to the reviewers’ comments point-by-point, as detailed below (our responses are in blue).

Reviewer 1

Here, authors took an innovative approach to characterize behavior and neural activity of naturalistic arm movements in people with epilepsy. Their rationale was that this approach is feasible when ECoG is used with conjunction of motion capturing. Their results strengthen the notion that this analysis is feasible and may shed light towards the neural and motor control of unstructured and spontaneous arm movements. Furthermore, their results stress out the importance of looking at the inter-subject and across events variability, which was used to model certain features. Among all features, only two strongly explained this variability. Taken together with this innovative approach, the findings of the present study can be used to test similar tasks in other clinical cohorts.

1) Authors should revise the text for typos etc (ex. Line 376: “cold” should be “could”). Thank you for catching this typo. We have corrected that typo (Line 388) and inspected

the rest of the manuscript for any other typos.

2) Authors should add a section about future directions/clinical implications. In this section they can talk what the next steps could be and how this approach can be possibly used in other clinical cohorts. Also, authors should discuss whether this approach can be used to test naturalistic movements with lower extremity.

Thank you for this suggestion. We have added the requested section on future directions and clinical implications in the penultimate paragraph of the discussion section (Lines

380-386):

Our results underline the importance of studying naturalistic movements and understanding neural variability across multiple days. Our approach leverages pre-existing clinical setups and could be extended to other movements and behaviors, such as grasping objects, sleep/wake transitions, and conversing with others. Future setups with multiple RGB or depth cameras would allow for improved motion tracking performance and the ability to analyze more complex 3D trajectories (Desmarais et al., 2020;

Mathis et al., 2020). In addition, our approach could be extended beyond epilepsy monitoring to study neural correlates of naturalistic movements during long-term scalp EEG recordings or chronic neural implants in other clinical cohorts (Duun-Henriksen et al., 2020; Gilron et al., 2020). It is also worth noting that such markerless pose tracking has also been used to track lower limb movements (Cao et al., 2019; Chambers et al., 2019), provided the subject remains within view of the cameras.

3) In the limitation section, authors should acknowledge the fact that the behavior was assessed while they were confined to a hospital bed. Therefore, these actions are not

100% naturalistic, but the arm movements are spontaneous and not constrained by

experimental factors (e.g., movement time, target size and location).

This is an excellent point and worth reiterating in our discussion. We have included this limitation of our study in the limitation paragraph of the discussion section (Lines 368-

370):

In addition, we assessed behavior from subjects who were confined to a hospital bed and selected parabolic movement trajectories for further analysis. Thus, these movements were not 100% naturalistic, but the arm movements we analyzed were still spontaneous and unconstrained by experimental factors.

4) Similar to the comment above, authors should consider revising the title. The word “naturalistic” may not be appropriate or accurate. They should consider replacing this word with “spontaneous”.

We discussed this point and considered revising the title, but we feel that “naturalistic” best describes the types of movements we were looking at. Even though the behavior we study does not reflect the full range of human behavior, these movements are still unconstrained, unstructured, and spontaneous. Therefore, we feel that “naturalistic” most appropriately describes these types of movements and would like to keep this wording in our title.

Reviewer 2

The manuscript addresses the considerable challenge of explaining the neural basis of movement by analyzing ECoG recordings during naturalistic movement performance. Unlike most common experiments where the results are performed in the controlled conditions of numerous repetitions of the same movement under the same precise conditions, here the authors carried out a series of experiments aimed at recording and analyzing thousands of natural arm movements, together with ECoG recordings from epilepsy patients.

I believe that the manuscript addresses two of the major problems of the study of motor control: the lack of natural conditions and the necessity of providing movement correlates in humans. Towards this end, the authors perform a careful study addressing two of the hard points in these kinds of problems: identifying relevant features both of behavior and of neural data.

One of the major challenges of analyzing unconstrained movements is their intrinsic variability. Amplitudes, orientations and targets are not defined, which turns movement characterization into a formidable task.

The authors characterized thousands of spontaneous movements enabling scalable analysis of video that was acquired simultaneously with the intracranial recordings. We characterized the variability of both naturalistic upper-limb reaching movements and the corresponding changes in cortical spectral power.

Their hypothesis is that naturalistic reaches would be associated with transient low- frequency power decreases and high-frequency power increases, localized from fronto- parietal sources. They also assessed the variability of these event-related spectral

power fluctuations and performed regression to partially explain this neural activity using behavioral frames.

Issues

I think their approach is courageous. However, my major concern is that the manner in which they select movement related features is unprincipled. In particular, movements were characterized not with a motion tracking system but rather from video annotation, which has to be done manually. A justification of the kinds of motor features you used should be provided.

Thank you for your feedback. First, we want to make clear that our pose estimation from video used automated, scalable computer vision techniques that we developed for this

purpose (as detailed in Methods). In particular, we estimated locations of joints using a neural network model (DeepLabCut) and then extracted movement events from these trajectories. While markerless pose estimation from video is not as accurate as commercially available motion tracking systems, these approaches have been very widely used because they greatly expand our capacity to study variability in natural behaviors (Mathis et al. Neuron 2020), including the opportunistic clinical dataset we have used here. The motor features we used were purposefully designed to capture this variability and are well connected to the literature (see more detailed justification below).

While some initial manual annotation was needed to train each DeepLabCut pose tracking model, the trained model automatically identified pose trajectories from all other video frames (only ∼0.01% of all video frames were annotated for each subject).

We have revised our description of the pose tracking models to clarify this point (Lines

110-112):

Note that these annotated frames comprised ∼0.01% of all video frames for each subject, demonstrating the infeasibility of annotating all video frames by hand.

In addition, we have added more citations justifying the motor features used to identify movements and the selected behavioral features used for the regression model (Lines

159, 118, and 124).

The features were extracted ad hoc, to quantify variations in movement parameters and environmental contexts. These features were later used as input variables for

regression models of inter-event spectral power and can be divided into 4 categories: Timing, Reach movement, Environmental, bimanual reach features.

- Wrist movements were labelled manually, but some criteria to select movement events were clearly ad hoc. Why can wrist movements only be parabolic? If the target is to analyze naturalistic movements, imposing this non-trivial constraint seems to defeat the purpose.

Thank you for bringing up this important point. We used a parabolic constraint to retain movements that could be well characterized for behavioral features such as reach angle and reach magnitude. We have included this parabolic constraint as a limitation in the discussion section (Lines 368-370):

In addition, we assessed behavior from subjects who were confined to a hospital bed and selected parabolic movement trajectories for further analysis. Thus, these movements were not 100% naturalistic, but the arm movements we analyzed were still spontaneous and unconstrained by experimental factors.

We chose parabolic wrist movements because they have been shown to be the building blocks used by the motor system to perform more complex trajectories (Polyakov et al. Biol Cybern 2009, added in Line 124) and can still reflect a variety of naturalistic reaching movements. Non-parabolic trajectories also often reflected false positive movements or brief movements with noisy tracking. We have also included a section in the penultimate paragraph of the discussion section explaining how future setups could use more cameras to improve pose tracking and enable analysis of cleaner, more complex movement trajectories:

Future setups with multiple RGB or depth cameras would allow for improved motion tracking performance and the ability to analyze more complex 3D trajectories (Desmarais et al., 2020; Mathis et al., 2020).

- With regard to the ECoG signals (I am agnostic as to this kind of signal), but why do you only use electrode power alone? Furthermore, you do not specifically explain how you coped with inter-subject variability in so far electrodes are placed differently.

We used electrode power for the regression model because it allows us to isolate high frequency activity that has previously been shown to be tied to movement (Holdgraf et al. Front Syst Neurosci 2017, Wagner et al. J Neurosci 2016, Miller et al. J Neurosci 2009). Additionally, our behavioral features were created such that we obtained only one value per event for each feature, so we needed a neural feature that can be averaged over timepoints. These reasons make frequency power an ideal measure compared to time- domain analyses and phase information.

To assess multi-subject neural activity with variable electrode placement, we projected electrode power onto common brain regions as described in the ’Projecting power into regions of interest’ subsection of the methods section. By aligning onto common brain regions, we are able to estimate inter-subject variability in a specific brain region (Fig. 5). This projection method assumes that neural activity occurs in similar brain regions across subjects, which seems reasonable for an upper-limb motor task (Miller et al. J Neurosci

2007).

- The reach movement feature extraction seems pretty much ad hoc. How did you correct for focal differences between images of different days / subjects?

This is an important point and one that we tried hard to address in our analytic methods. Because these recordings were opportunistic and took place in a clinical setting, only the clinical staff was allowed to move the camera. We minimized the effect of focal differences by selecting movement features that were relative to the start of each reach. This limitation of our analysis is included in the discussion section (Lines 374-377):

Additionally, the video camera was positioned by the clinical staff and was vertically rotated away by the clinical staff during private times, meaning that the video view changed slightly throughout the day. We minimized the effect of such rotations on our behavioral features by excluding camera rotation events and using movement features that were relative to the start of each reach.

- The section of environmental features is ambiguously described. However, one possible method of validation is correlation or a metric of similarity between them.

- The variability across subjects is within norm, but I would expect you to validate the specific features by statistically comparing across subjects.

Thank you for these excellent two points. We have added a statistical comparison across subjects for each feature using a one-way Kruskal-Wallis test, along with statistical tables for each of the 10 behavioral and environmental features reporting the 95% confidence interval for every subject (Tables 2-1-2-2). We included the results from these statistical tests in our description of behavioral features in Fig. 2 to provide statistical validation of inter-subject variability (Lines 247-249). In addition, we have performed correlations between features that you suggested in Fig. 2-2 (Lines 260-270):

All ten metadata features significantly differed across subjects (p<0.001 for every feature, one-way Kruskal-Wallis test). See Tables 2-1-2.2 for further statisticaldetails.

- What specific criterion did you use to organize your frequency separation?

We used the 8-32 Hz and 76-100 Hz frequency bands based on the findings of Miller et al. (2007). These same frequency bands have since been used by several additional studies in comparable circumstances. We have added this citation when describing the dependent variable used for regression (Lines 198-199).

- You need to explain better the kinds of models you use for single-event spectral power with behavioural features (expand on FIG 6a). Is it just a linear model?

That is correct, we used a linear regression model, with a Huber-norm loss to minimize the effects of outliers. We have revised Fig. 6a to make this more clear.

Minor Issues

- The presentation of events is very hard to read (FIG5)

Thank you for pointing out this issue. We have stacked the panels of Fig. 5 vertically instead of horizontally to improve readability.
